# Pain Obtunders

**Published:** 1886-09

**Authors:** A. W. Harlan

**Affiliations:** Chicago, Ill.


### PAIN OBTUNDERS.

BY A. W. HARLAN, M. D., D. D. S., CHICAGO, ILL.

READ BEFORE THE INDIANA STATE DENTAL SOCIETY.

It has long been the desire of every dentist to excavate cavities in teeth painlessly, but the fruition of that desire I fear has not yet arrived, except in pulpless teeth and such superficial cavities as are found in the fissures of bicuspids and molars. It has been stated on several occasions that, by drying a cavity and the use of a sharp excavator, all that is necessary for the obtunding of the dentine has been done, but such is not the fact in all cavities. I believe that it is essential to thoroughly dry cavities in general before much excavating has been done, in order to avoid producing pain, but complete dryness is not sufficient, not even when the air is continually heated.

It is not practicable to produce a continuous stream of heated air unless you have it compressed, or have a compressor attached to a steam heating apparatus, or have a foot-bulb which a water or other motor will put in motion, so as to have a steady stream. It is not certain that the use of heated air for any considerable length of time may not be injurious to a tooth, by driving off too much moisture and checking the enamel so seriously as to produce leakage around a gold filling. I think I have seen evidences of this in pulpless teeth, at any rate. The only practical method of using heated air for obtunding sensitive dentine is to direct the jet on the cavity for a half to a whole minute, and then introduce with a pipette or dropper the obtunding agent, which may be alcohol, ether, carbolic acid, in a generous solution, or other local anæsthetic. This should be continued for about two minutes, and in most cases the patient will not shrink when the excavator is used. The rubber dam must be used in every case. This method of using heated air is, I believe, entirely new and original with the writer. Every one present must remember some case where it seemed impossible at the time to properly prepare the cavity in a tooth so that a respectable operation could be performed. This method will undoubtedly help some one in just such cases. You cannot accomplish much by using an ordinary bulb-syringe. One of Glow & Young's air compressors, or C. Beseler's, or some other maker, whereby you can have a pressure of from forty to sixty pounds to the inch, will be all that is needed. If the heated air is directed from the cylinder, the pipe or taps must be protected with a non-conductor by having a coiled tube about eighteen to twenty-four inches from the mouth of the patient; a small gas jet or spirit lamp will produce all the heat needed. As the stream of air comes from the compressors through the heated pipe, you can have a small thermometer attached, and can easily regulate the degree of heat; that portion of the pipe nearest the mouth must be covered with a non-conductor, to protect the lips and face. A sheath of asbestos paper or bone can be used, so that it need not touch the heated pipe.

In the use of any agent for obtunding sensitive dentine, dryness is absolutely required. Time is another factor; I do not always feel that I can afford to wait for an obtunder to act, and many patients certainly cannot, therefore we seek for a rapid and safe method of depriving dentine of its sensibility. No doubt you are all acquainted with the most recent addition to the list of obtunding agents. This includes the various forms of Cocaine, of which I shall say nothing, Cannabis Indica, and the Oleo-resin of Kora-Kora. In times gone by I have experimented with and used the ethereal oils, separately and combined, carbolic acid crystals both cold and heated, cetolized potash and glycerine, chloride of zinc, sulphate of zinc, camphors, including menthol, hydrate of chloral, ether, alcohol, tincture of aconite root, and other vegetable tinctures, prepared chalk, and other antacids, iodoform, iodide of potassium, glycerine, chloroform, and many other agents, some of which were secret anæsthetics, and hence valueless. Occasionally some one of the above substances would prove of some value, but when most needed they could not be relied upon. The principal difficulty has been that obtunding agents either required too long a period of waiting to obtain results, or they were themselves productive of pain. The latter is particularly true of those drugs or agents that rapidly abstract water, or are escharotic. For these reasons the majority of the above cannot be relied upon for destroying sensibility in dentine.

Preparatory to the use of an obtunder the cavity should be opened with a chisel and the debris washed out with warm water, the rubber dam should then be adjusted, and if the necks of the teeth are sensitive or the gums tender, they ought to be painted or swabbed with the tincture of cannabis indica slightly warmed. The cavities should then be dried, after which pellets of cotton moistened with alcohol may be introduced and quickly removed, and the obtunder, whatever it may be, should then be placed in the cavity and allowed to remain while you are operating on some small fissure or other non-sensitive cavity. By so proceeding you inspire the patient with confidence, gain time for the drug to act, and allow the alcohol to evaporate, which is in itself a tolerable obtunder. By preference, and from confidence in the drug, I am still using the fluid extract of cannabis indica. I find that, used in the above manner, it acts more rapidly than any other known drug which is not injurious to the teeth. In previous papers I have cautioned against too free use of the fluid extract, as it is poisonous in large doses when accidentally used, one-half to one minim being the ordinary dose, while five to twenty minims of the tincture may be given with safety.

You will find that the tincture of cannabis indica and the fluid extract are extremely valuable in other directions than that of obtunding dentine, as you can open an abscess almost painlessly by pricking its surface two minutes before introducing the lancet. By soaking a pulp with the fluid extract for a few minutes you can remove it without pain, provided you have free access to it, so that the brush can be plunged into the canal without unnecessary fumbling around the entrance. You can use the tincture for injections around the root of a tooth, when there are fine deposits on the sides which would cause great pain in removal without its use. I habitually make great use of it with perfect results. I have extracted a few roots of teeth by injecting a drop on each side, and also painting the gums adjacent to the roots, and waiting for five minutes before removing them. The tincture should be warmed before using it for this purpose.

The Oleo-resin of Kora-Kora I have used for obtunding the soft tissue and dentine to a limited extent, but it is so disagreeable to use, being greasy and having a bad taste, that I am at present seeking for some method of preparing it so that it will not be so nauseating. You can get it from Parke, Davis & Co., and see what merit it possesses for the above purposes. . So far, I am certain that it is less efficient than cannabis indica. In conclusion, allow me to say that at present it is possible to prepare and fill cavities in teeth without the destruction of the pulp, which, in many cases before obtundents were properly used, it was impossible to fill with any degree of satisfaction to the operator, and often such operations were of little benefit to the patient.

